# Cytotoxic peripheral T-cell lymphomas and EBV-positive T/NK-cell lymphoproliferative diseases: emerging concepts, recent advances, and the putative role of clonal hematopoiesis. A report of the 2022 EA4HP/SH lymphoma workshop

**DOI:** 10.1007/s00428-023-03616-4

**Published:** 2023-08-30

**Authors:** Fina Climent, Alina Nicolae, Laurence de Leval, Stefan Dirnhofer, Lorenzo Leoncini, Sarah L. Ondrejka, Lorinda Soma, Andrew Wotherspoon, Alberto Zamo, Leticia Quintanilla-Martinez, Siok-Bian Ng

**Affiliations:** 1 Department of Pathology, Hospital Universitari de Bellvitge-IDIBELL, Feixa Llarga s/n, L’Hospitalet de Llobregat, 08907 Barcelona, Spain; 2 Department of Pathology, Hautepierre, University Hospital Strasbourg, Strasbourg, France; 3 Institute of Pathology, Department of Laboratory Medicine and Pathology, Lausanne University Hospital and Lausanne University, Lausanne, Switzerland; 4 Institute of Medical Genetics and Pathology, University Hospital Basel, University of Basel, Basel, Switzerland; 5 Department of Medical Biotechnology, Section of Pathology, University of Siena, Siena, Italy; 6 Pathology and Laboratory Medicine Institute, Cleveland Clinic, Cleveland, OH USA; 7 Department of Pathology, City of Hope National Medical Center, Duarte, CA USA; 8 Department of Histopathology, Royal Marsden Hospital, London, UK; 9 Institute of Pathology, University of Würzburg, Würzburg, Germany; 10 Institute of Pathology and Neuropathology, Eberhard Karls University of Tübingen and Comprehensive Cancer Center, University Hospital Tübingen, Tübingen, Germany; 11 Department of Pathology, Yong Loo Lin School of Medicine, National University of Singapore, Main Building, Level 3, 5 Lower Kent Ridge Road, Queenstown, Singapore; 12 Cancer Science Institute of Singapore, National University of Singapore, Main Building, Level 3, 5 Lower Kent Ridge Road, Queenstown, Singapore

**Keywords:** T-cell lymphoma, Cytotoxic T-cell lymphoma, Clonal hematopoiesis, NK-cell lymphoma, Epstein–Barr virus, EA4HP workshop

## Abstract

Cytotoxic peripheral T-cell lymphomas and EBV-positive T/NK-cell lymphoproliferative diseases were discussed at the 2022 European Association for Haematopathology/Society for Hematopathology lymphoma workshop held in Florence, Italy. This session focused on (i) primary nodal EBV-positive T and NK-cell lymphomas (primary nodal-EBV-TNKL), (ii) extranodal EBV-positive T/NK lymphoproliferative diseases (LPD) in children and adults, (iii) cytotoxic peripheral T-cell lymphomas, NOS (cPTCL-NOS), EBV-negative, and (iv) miscellaneous cases. Primary nodal-EBV-TNKL is a newly recognized entity which is rare, aggressive, and associated with underlying immune deficiency/immune dysregulation. All cases presented with lymphadenopathy but some demonstrated involvement of tonsil/Waldeyer’s ring and extranodal sites. The majority of tumors are of T-cell lineage, and the most frequent mutations involve the epigenetic modifier genes, such as *TET2* and *DNMT3A*, and JAK-STAT genes. A spectrum of EBV-positive T/NK LPD involving extranodal sites were discussed and highlight the diagnostic challenge with primary nodal-EBV-TNKL when these extranodal EBV-positive T/NK LPD cases demonstrate predominant nodal disease either at presentation or during disease progression from chronic active EBV disease. The majority of cPTCL-NOS demonstrated the TBX21 phenotype. Some cases had a background of immunosuppression or immune dysregulation. Interestingly, an unexpected association of cPTCL-NOS, EBV-positive and negative, with TFH lymphomas/LPDs was observed in the workshop cases. Similar to a published literature, the genetic landscape of cPTCL-NOS from the workshop showed frequent mutations in epigenetic modifiers, including *TET2* and *DNMT3A*, suggesting a role of clonal hematopoiesis in the disease pathogenesis.

**Supplementary information:**

The online version contains supplementary material available at 10.1007/s00428-023-03616-4.

## Introduction

Cytotoxic T lymphocytes (CTLs) and natural killer (NK) cells represent subsets of immune cells with a major role in host epithelial immune surveillance. Both populations display a similar approach of target cell “killing” by releasing granule-associated cytotoxic proteins into the immunological synapse. However, their mechanism of target recognition is distinct, allowing for their complementarity in ensuring host defense [1]. The CTLs are mostly represented by alpha/beta CD8+ T cells and in minor proportion by gamma/delta T cells and CD4+ T cells [2, 3].

Mature T- and NK-cell neoplasms displaying cytotoxic phenotype are uncommon and highly heterogeneous, with 21 entities described by the 5th edition of the WHO classification and the 2022 International Consensus Classification (ICC) [4, 5]. This diversity likely reflects the array of normal cytotoxic cells, their functional plasticity and differentiation state, disease localization, and association with pathogens [e.g., Epstein-Barr virus (EBV)]. They are commonly extranodal, following the normal distribution of cytotoxic lymphocytes with few entities presenting primarily in the lymph node (LN). The improved knowledge of T-cell ontogeny combined with integrated genomic and transcriptomic approaches have led to a better understanding of the putative cell-of-origin for some of these entities. Although most cytotoxic T-cell lymphomas are highly aggressive, broadly speaking the cytotoxic phenotype alone cannot accurately predict a patient’s outcome. Some T- and NK-cell lymphoproliferative disorders follow an indolent clinical course and a subset of them may progress/transform to aggressive diseases [e.g., localized/indolent forms of chronic active EBV disease (CAEBVD) and T-cell large granular lymphocytic leukemia (T-LGL)] [6–9].

Session 4 of the Lymphoma Workshop (LYWS) organized by the European Association for Haematopathology and Society for Hematopathology (EA4HP-SH) in Florence September 2022 was dedicated to cytotoxic T-cell lymphoma (excluding skin) and EBV-positive nodal T/NK-cell lymphoma (excluding extranodal NK/T cell lymphoma, nasal type). A total of 35 cases were reviewed by the expert panel members, and in this paper, they were grouped into the following categories: (1) Primary nodal EBV-positive T/NK-cell lymphoma, (2) extranodal EBV-positive T/NK lymphoproliferative disorders (LPD) in childhood and adults, (3) cytotoxic T-cell lymphoma, EBV-negative (4) miscellaneous cytotoxic PTCL, and (5) findings from the workshop.

Based on the submitted cases, this workshop report aims to discuss and summarize the distinctive features of primary nodal EBV+ T/NK-cell lymphoma and the role played by the underlying immune deficiency/impairment and clonal hematopoiesis (CH) in cytotoxic TCL biology and their potential progression from an indolent T-LPD. Furthermore, the novel and unusual association of cytotoxic TCL with TFH LPDs/lymphomas will be addressed.

## EBV-positive nodal T- and NK-cell lymphoma (primary nodal EBV+ T/NK-cell lymphoma)

Nodal EBV-positive T- and NK-cell lymphoma (primary nodal-EBV-TNKL) is now recognized as a distinct entity in 5th edition of the WHO lymphoma classification; previously, it was subsumed as a subtype under the entity of PTCL-NOS [4]. In the 2022 ICC, it is listed as a provisional entity and termed “primary nodal EBV-positive T/NK-cell lymphoma” to highlight the primary nodal disease origin and to distinguish them from other T/NK EBV+ LPDs that may infiltrate predominantly lymph nodes [5]. This disease is rare and occurs mostly in older adults from East Asia [10–15]. The tumor is mostly of T-cell lineage and is characterized by frequent loss of 14q11.2, and upregulation of immune pathways, NFκB and PD-L1 [15, 16]. Most cases show type II EBV latency pattern. The disease has an aggressive behavior with median overall survival ranging 2.5–8.0 months [12–15, 17]. Despite its aggressiveness, the tumor demonstrates lower genomic instability compared to extranodal NK/T-cell lymphoma (ENKTL), nasal type, and PTCL-NOS [16].

A total of 8 cases, 5 females and 3 males were submitted to the workshop (Supplementary Table 1). The age ranged from 46 to 81 years (median 54.5 years). Five patients were Asians, and the remaining 3 were Caucasians. Consistent with the literature, an association with underlying immune deficiency or conditions which may impair immune responses was present in some cases, including HIV (*n* = 1), hepatitis B (*n* = 2), and prior history of angioimmunoblastic T-cell lymphoma (AITL) (*n* = 2, cases LYWS-1190 and LYWS-1396) [17–19]. Case LYWS-1190 from RKH Au-Yeung was a typical example of primary nodal-EBV-TNKL occuring in a 46-year-old female who had a prior history of AITL 2 years ago. Case LYWS-1396 submitted by Wang L occurred in a patient with AITL diagnosed in 2012 and subsequently developed primary nodal-EBV-TNKL in 2019 which was clonally unrelated to the AITL (see section 6.1 for further discussion).

All the cases presented with lymphadenopathy, but some cases additionally demonstrated other sites of involvement, including spleen (*n* = 1), tonsil (*n* = 1), and extranodal sites such as pleural effusion (*n* = 1), skin (*n* = 1), and lacrimal glands (*n* = 1). Notably, nasal disease was not detected. The involvement of the tonsil/Waldeyer’s ring in rare cases of primary nodal-EBV-TNKL may be interpreted as upper aerodigestive tract involvement and be mistaken for ENKTL. This is illustrated by LYWS-1207 submitted by K. Ofori, a 67-year-old Caucasian female with extensive lymphadenopathy and involvement of the spleen, tonsil, and lacrimal glands. The tumor demonstrated monoclonal TR gene rearrangement and mutations of *TET2* and *DNMT3A*, which are uncommon in ENKTL. In the study from Wai CMM et al., 4 of 25 cases of primary nodal-EBV-TNKL presented primarily with nodal disease and also displayed tonsil/Waldeyer’s ring involvement [16]. In all 4 cases, the tumors were of T-cell origin and 3 demonstrated *TET2* and/or *DNMT3A* mutations (supplementary table 1). Interestingly, Nicolae et al. described 7 cases of EBV-positive cytotoxic PTCL and 3 cases had “Ear-Nose-Throat” involvement, although it was uncertain if the nasal site was involved by tumor [20]. These 3 cases revealed both *TET2* and *DNMT3A* mutations. It is worth noting that the tonsils, Waldeyer’s ring, and spleen are considered nodal tissue, not extranodal tissues, according to the Lugano classification and staging of lymphoma [21]. Therefore, the involvement of the tonsil/Waldeyer’s ring does not necessarily exclude the diagnosis of primary nodal-EBV-TNKL. Similarly, primary nodal-EBV-TNKL should also be distinguished from ENKTL with nodal involvement. In such cases, a thorough assessment of clinical, histopathological, and molecular features is necessary to distinguish primary nodal-EBV-TNKL involving extranodal sites and tonsil/Waldeyer’s ring from ENKTL with nodal involvement. The presence of primary nodal disease with tumors involving mainly lymph nodes, T-cell lineage, the absence of nasal disease, and the presence of epimutations would favor the diagnosis of primary nodal-EBV-TNKL over ENKTL.

**Fig. 1:**
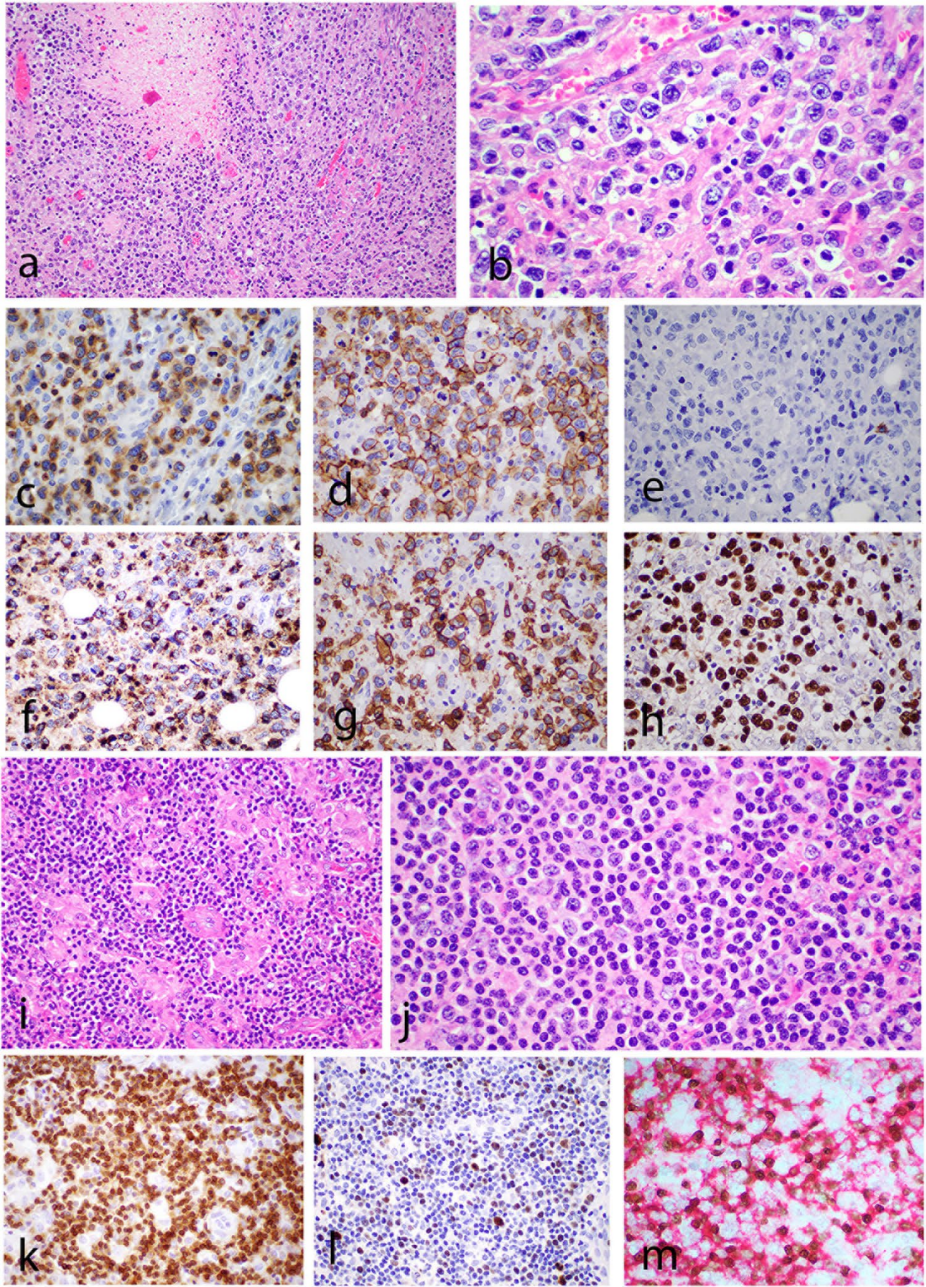
Histologic features of nodal EBV-positive T and NK-cell lymphoma. **a**–**f** Case LYWS-1138, courtesy of L. Goh. **a** The tumor shows areas of necrosis and diffuse sheets of neoplastic cells. **b** The tumor cells are large with irregular vesicular nuclei, coarse chromatin, and prominent nucleoli. Immunohistochemistry reveals positive expression for **c** CD3, **d** CD8, **f** TIA1, **g** TCRgamma, and **h** EBER and negativity for **e** CD56. **i**–**m** Case LYWS-1190, courtesy of R.K.H. Au-Yeung. **i** The tumor reveals predominantly small cells with abundant histiocytes in the background, resembling lymphoepithelioid (Lennert) lymphoma. **j** Tumor cells display small monotonous nuclei with mild nuclear atypia and indistinct nucleoli. They are positive for **k** TCRβF1. **l** Ki67 proliferation index is low. **m** EBER/CD8 double stain demonstrates that the neoplastic cells are positive for CD8 and EBER

Six of the 8 cases submitted demonstrated medium to large tumor cells, and one case had a mixed small to large cell morphology. In the case LYWS-1138 submitted by L Goh, the tumor cells were large and showed CD8+/CD56−/TCRgamma+ phenotype (Fig. 1A–H). LYWS-1190 submitted by RKH Au-Yeung demonstrated a tumor composed of small cells with abundant histiocytes in the background, resembling lymphoepithelioid (Lennert) lymphoma, and a low Ki-67 proliferation index (Fig. 1I–M). A rich inflammatory background was present in three cases and necrosis is seen in two cases. Unlike ENKTL, an angiocentric growth was only present in 1 out of 8 cases. Case LYWS-1176, submitted by Y Zhang, represented an unusual example of a composite primary nodal-EBV-TNKL and classic Hodgkin lymphoma (CHL).

Phenotypically, all eight cases were positive for CD3 and/or CD2 and demonstrated an activated cytotoxic phenotype. CD56 expression was negative in 6 out of 8 cases. Most cases (6 of 8) were positive for CD8 while CD4 was negative in all cases except for one. A recent study comparing primary nodal-EBV-TNKL and ENKTL reported that the CD8−/CD56+ phenotype is associated with NK-cell lineage while the CD8+/CD56− phenotype is associated with T-cell origin [15]. Therefore, the expression of CD8 and CD56 can provide a clue to the T vs NK lineage especially when clonality testing is not available.

Based on a combination of positive expression of TCR alpha/beta and/or TCR gamma/delta using immunohistochemistry (IHC) and/or monoclonal TR gene rearrangement, 6 out of the 7 cases analyzed show T-cell lineage. NK-cell origin is defined as the absence of IHC expression of TCR alpha/beta and TCR gamma/delta, absence of clonal TR gene rearrangement, and frequent expression of CD56. Only one case (14%), LYWS-1227 from C Bárcena, was likely of NK-cell lineage as the tumor revealed negative expression for CD4, CD8, CD56, TCR alpha/beta, and TCR gamma/delta and was also polyclonal for TR gene rearrangement. Of the 6 cases tested by IHC, 3 expressed TCR alpha/beta, one expressed TCR gamma/delta, and the remaining were silent for TCR alpha/beta and TCR gamma/delta.

**Fig. 2:**
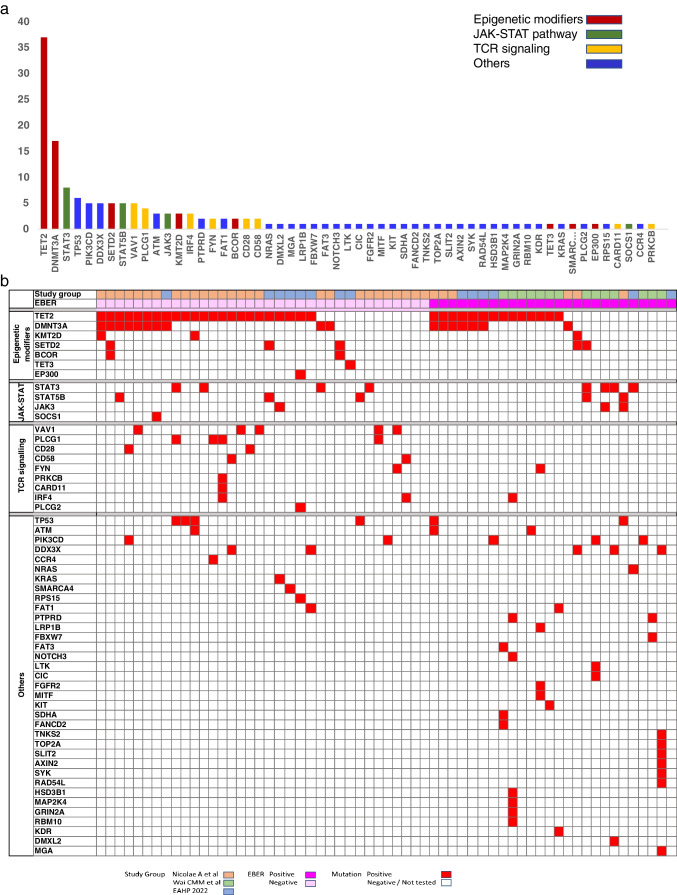
Mutational landscape of nodal cytotoxic peripheral T-cell lymphomas (PTCL) based on workshop cases and cases from recent literature with next generation sequencing data (Wai CMM. Haematologica (2022) 1;107(8):1864, Nicolae A. Modern Pathology (2022) 35:1126–1136). Histogram plot (**a)** and heatmap (**b**) illustrate the frequency of mutations in all cases of cytotoxic PTCL, both EBV-positive and EBV-negative. A total of 61 cases analyzed revealed that the most common mutations involve epigenetic modifiers, such as TET2 and DNMT3A, JAK/STAT pathway and TCR signaling genes

Next generation sequencing (NGS) data were available in 6 of the 8 workshop submitted cases and revealed mutations of epigenetic modifier genes, such as *TET2* (*n* = 4), *DNMT3A* (*n* = 3) as well as *STAT3* (*n* = 1), *NRAS* (*n* = 1), and *PIK3CD* (*n* = 1). The panel subsequently analyzed mutation data of 26 cases of primary nodal-EBV-TNKL, including the 6 workshop cases and 20 cases published in the literature [16, 20]. The most frequent mutations involve the epigenetic modifier genes, such as *TET2* (14/26, 54%), *DNMT3A* (7/26, 27%), *SETD2* (2/26, 8%), and *KMT2D* (1/26, 4%). JAK/STAT pathway genes were mutated as follows: *STAT3* (4/26,15%), *STAT5B* (2/26, 8%), and *JAK3* (2/26, 8%). Other mutations include *PIK3CD* (3/26, 12%) and *DDX3X* (3/26, 12%) (Fig. 2). The mutational landscape of primary nodal-EBV-TNKL is consistent with that observed in EBV-negative nodal cytotoxic PTCL [20] suggesting a putative role of clonal hematopoiesis (CH) in this rare lymphoma.

An issue raised during the workshop but remains unresolved was the biologic relationship between primary nodal-EBV-TNKL and EBV-negative cytotoxic PTCL NOS (cPTCL-NOS). Preliminary data suggests that primary nodal-EBV-TNKL has worse outcome and lower genomic instability compared to EBV-negative cPTCL-NOS [16]. However, their mutational profiles are similar with frequent mutations of epigenetic modifier genes. Further studies are needed to clarify if these 2 entities are indeed related.

There is currently limited data on the treatment of this rare and aggressive disease. The outcome of patients treated with etoposide and chemotherapy regimens with and without anthracycline is poor [14, 22, 23]. It remains uncertain if patients with nodal-EBV-TKNL will show a similar favorable response to L-asparaginase-based regimens, such as SMILE (steroid, methotrexate, ifosfamide, l-asparaginase, and etoposide) as observed in patients with advanced ENKTL [24]. Overexpression of PD-L1 has been reported in ENKTL [25], and anti-PD1 immunotherapy has been shown to be effective in patients with relapsed and refractory ENKTL [26]. Interestingly, PD-L1 protein is significantly overexpressed in both tumor and non-tumor cells in primary nodal-EBV-TNKL compared to ENKTL, and this PD-L1 upregulation in primary nodal-EBV-TNKL may have potential therapeutic implications for anti-PD1 treatment [16].

In conclusion, the key take-home messages for primary nodal-EBV-TNKL from the workshop are summarized below:

1. Commonly primary nodal-EBV-TNKL is associated with underlying immune deficiency or conditions which may impair immune responses.
2. Primary nodal-EBV-TNKL shows frequent mutations of epigenetic modifier genes, including *TET2* and *DNMT3A*, suggesting a possible role of CH.
3. Primary nodal-EBV-TNKL can involve extranodal sites and, less commonly, the Waldeyer’s ring and should be distinguished from ENKTL with nodal disease. In such cases, it is essential to perform a thorough assessment of clinical, histopathological, and molecular features to distinguish them from ENKTL with nodal involvement. The primary nodal presentation with tumor mainly involving lymph nodes, T-cell origin, lack of nasal involvement, and presence of mutations in *TET2* and *DNMT3A* supports the diagnosis of primary nodal-EBV-TNKL.

## EBV-positive extranodal T/NK-cell lymphoproliferations

**Fig. 3:**
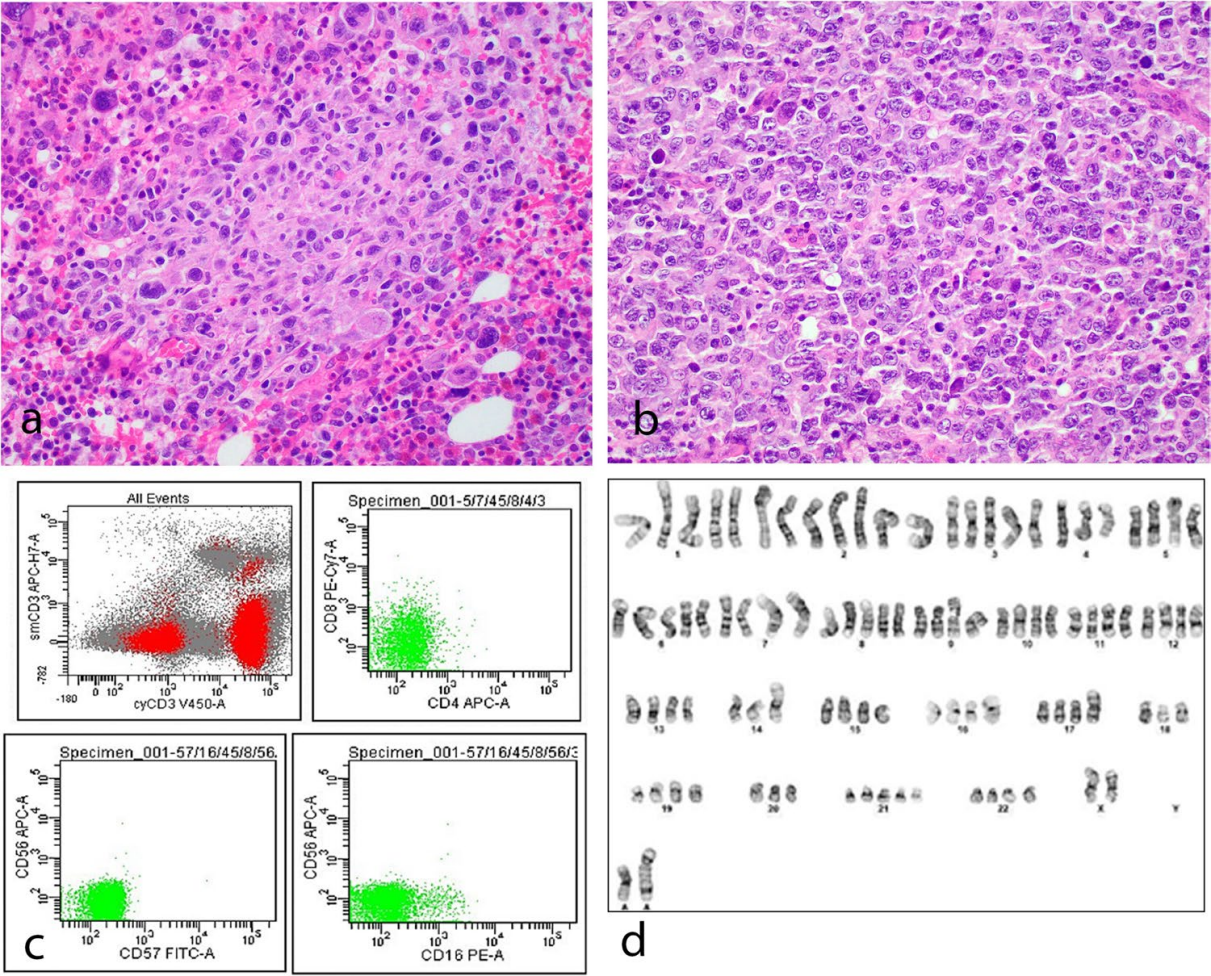
Case LYWS-1181 submitted by I Obiorah was an example of aggressive NK-cell leukemia (ANKL). The patient presented with multiple lymphadenopathy, fever, HLH, and involvement of the BM and spleen. The tumor cells in the BM (**a**) and LN (**b**) were large and pleomorphic. Flow cytometry (**c**) revealed an infiltrate which was cCD3+, sCD3−, CD4−, CD8−, CD56−, CD57−, and CD16−. Cytogenetics demonstrated hyperploidy with multiple tetraploid clones and complex cytogenetic anomalies (**d**)

A total of 9 cases of EBV-positive T/NK-cell LPD involving extranodal sites in children and adults were submitted to the workshop (Supplementary Table 2). Three cases demonstrated prominent nodal involvement either at disease presentation or during transformation and illustrate the potential diagnostic difficulty with primary nodal-EBV-TNKL. The first case, LYWS-1087 submitted by L Lorenzi, represented a case of severe mosquito bite allergy (SMBA) with secondary progression to an aggressive NK-cell leukemia (ANKL) showing prominent lymph node involvement. The second case (LYWS-1440 submitted by CV Curry) is an example of systemic EBV-positive T-cell lymphoma of childhood (SEBVTCL) with predominant lymph node disease at presentation but otherwise typical manifestations of hemophagocytic lymphohistiocytosis (HLH) and elevated EBV DNA titers in a 2-year-old Hispanic boy. Both cases illustrate that EBV-positive T/NK LPD involving extranodal sites, such as ANKL and SEBVTCL, can occasionally show prominent nodal disease at presentation or following transformation from systemic or localized forms of CAEBVD and should not be misdiagnosed as primary nodal-EBV-TNKL, a disease of adults and elderly patients [27–29]. The third case, LYWS-1181 submitted by I Obiorah, was a challenging example of ANKL. The patient was a 46-year-old African man presenting with extensive lymphadenopathy, fever, HLH, and involvement of the BM and spleen by an EBV-positive cytotoxic T/NK infiltrate which was cCD3+, sCD3−, CD7+, CD4−, CD8−, CD56−, TCRalpha/beta−, and TCRgamma/delta− (Fig. 3). TR gene rearrangement showed a polyclonal pattern. The tumor revealed a complex karyotype (Fig. 3D) and NGS identified mutations of *IDH2* and *LRP1B*, which are not commonly present in ANKL. The phenotype favored an NK-cell lineage based on the absence of expression of T-cell markers, but there was a lack of expression of NK-cell markers, including CD56 and CD16. The panel found it challenging to distinguish ANKL from primary nodal-EBV-TNKL and favored the former in view of the presence of BM involvement, HLH at presentation and a complex karyotype.

LYWS-1327 from S Sánchez was an interesting example of a systemic EBV+ T-cell LPD occurring in a patient with primary immunodeficiency. The patient is a 12-year-old girl who presented with pancytopenia, fever, hepatosplenomegaly, and progression to HLH with multiple enlarged LN. She underwent allogeneic BM transplantation 7 months later. Subsequent analysis identified a G109S mutation of the *TNFRSF9/4-1BB*, which resulted in defective CD8+ T-cell activation and cytotoxicity against EBV-infected B cells in vitro. Deficiency of 4-1BB is associated with EBV-positive B-cell proliferation, Hodgkin lymphoma, and chronic active EBV disease (CAEBV) of T-cell type [30, 31]. Although reported in the literature, the term CAEBVD should not be used in the context of immunodeficiency, and therefore, a descriptive term such as systemic EBV+ T-cell LPD is preferable in this case. There were episodes of EBV reactivation, but the patient was otherwise clinically well at the last follow up 10 years later.

Two cases submitted in this group illustrated the diagnostic challenge between CAEBVD and a self-limiting EBV+ LPD or infectious mononucleosis (IM) with protracted clinical course [32]. One case occurred in an elderly patient (LYWS-1126 from X Huang) who presented with lymphadenopathy, HLH, elevated EBV-IgM, and EBV DNA titer. An EBV-positive, CD8-positive, polyclonal T-cell proliferation was present in the LN, spleen, and BM. The patient required anti-viral treatment for 1.5 years and remained well 3.5 years later. Based on the elevated EBV-IgM and IgG levels, it is likely that the patient initially had acute EBV infection or EBV reactivation [33], which subsequently developed a protracted clinical course. Since a double stain for EBER/CD79a and EBER/CD3 was not performed to confirm the lineage of the EBV-infected cells, the panel acknowledged the submitter’s diagnosis that a mild form of CAEBVD cannot be excluded due to the prolonged clinical course. A second case of a self-limiting EBV-associated LPD was also submitted by L Zhang (LYWS-1348). This occurred in a 21-year-old man with lymphadenopathy and splenomegaly. A cytotoxic and polyclonal CD8+ T-cell proliferation was present in the lymph node and spleen. The patient’s symptoms resolved spontaneously after 4 months without treatment, which is unusual for CAEBVD. There were no manifestations of HLH, which makes the diagnosis of EBV-associated HLH unlikely. The panel acknowledged the differential diagnosis of IM and CAEBVD at the time of initial diagnosis. To help distinguish between the two, a double stain for EBER with CD3 and CD20 or CD79A would have been needed, as EBV typically infects B cells and rarely CD8+ T cells in IM [34] and T or NK cells in CAEBVD. The T cells in CAEBVD are more often CD4-positive while those in IM are often CD8-positive [32, 35]. Additionally, the positive expression of LMP1 and EBNA2 supports the diagnosis of IM over CAEBVD. However, due to the limited material available for further workup and the small number of EBER-positive cells present in this case, the panel favored a self-limiting EBV+ LPD, most likely IM.

During the workshop, the panel discussed the differential diagnosis of EBV-positive T/NK LPD (Table 1) and reached several important conclusions:

1. Depending on the T- or NK-cell lineage, CAEBVD can progress to more a aggressive EBV+ T/NK-cell lymphoma or leukemia, such as SEBVTCL, ENKTL, or ANKL; these aggressive diseases should not be diagnosed as primary nodal-EBV-TNKL.
2. Both SEBVTCL and ANKL can have prominent lymph node involvement, either at presentation or following transformation/progression from localized/indolent forms of CAEBVD and should not be mistaken as primary nodal-EBV-TNKL. The presence of systemic (leukemic) disease and HLH distinguish SEBVTCL and ANKL from primary nodal-EBV-TNKL. In addition, an NK-origin, leukemic disease and/or BM involvement, and complex karyotype will favor ANKL over primary nodal-EBV-TNKL.
3. The majority of IM is self-limiting, and patients usually recover without complications. These cases do not pose diagnostic challenges with CAEBVD. Less commonly, IM can develop a protracted course lasting more than 3 months and may be mistaken for CAEBVD. In this context, it is important to determine the lineage of the EBV-infected cells. Positive expression of LMP1 and EBNA2 stains is also helpful and favors IM over CAEBVD.
4. Although a small proportion of T cells, often CD8 positive, can be infected with EBV in IM [34], the majority of EBV-positive cells represent B cells, unlike CAEBVD where the majority of EBV-infected cells are T or NK cells. The self-limiting clinical course and resolution of symptoms also favor IM over CAEBVD.

**Table 1:**
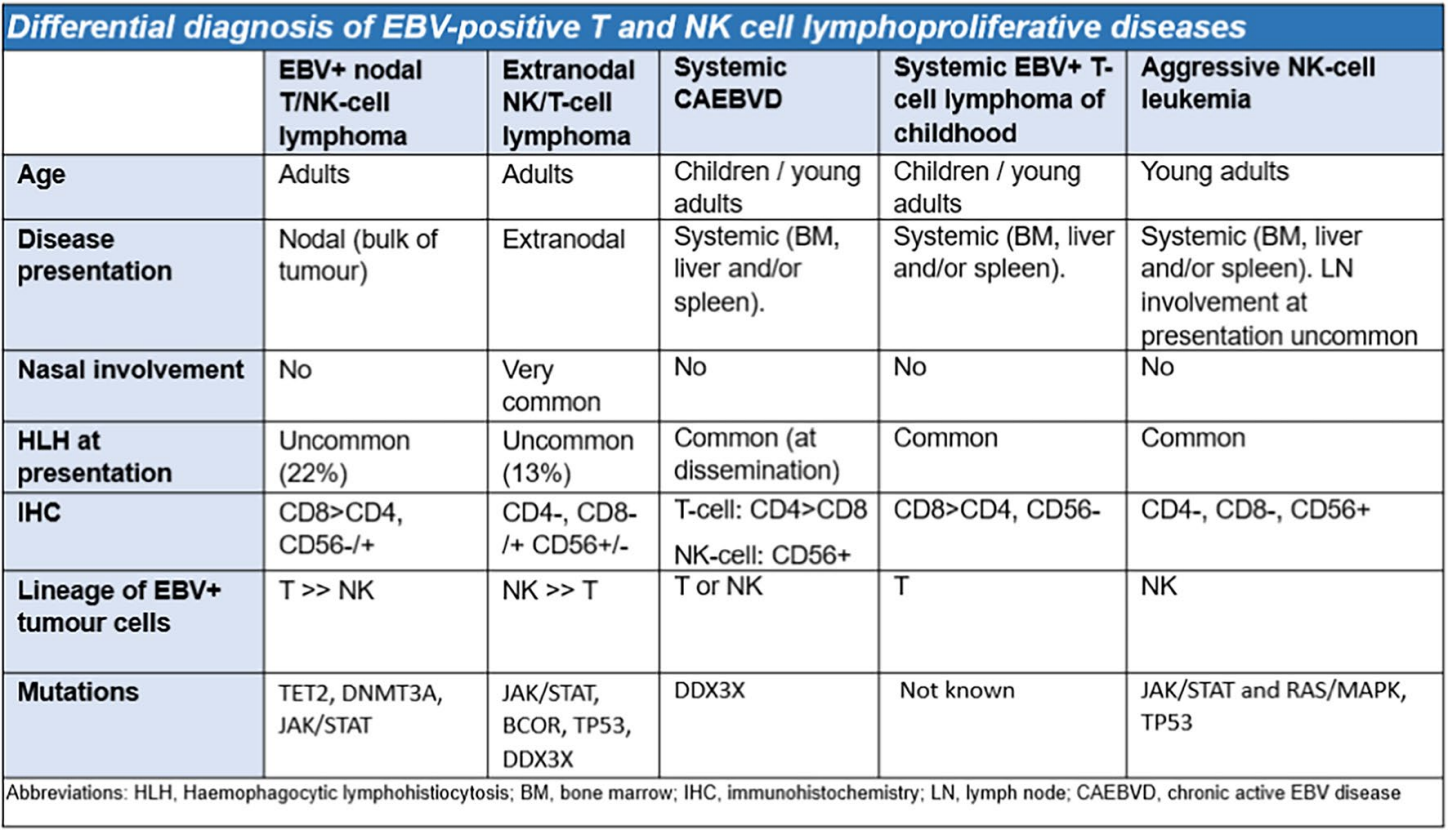
Differential diagnosis of EBV-positive T and NK-cell lymphoproliferative diseases

## Cytotoxic PTCL-NOS, EBV-negative

As described in a recent paper [20], cytotoxic PTCL-NOS (cPTCL-NOS), is defined by the expression of at least one cytotoxic molecule in more than 50% of tumor cells and they frequently present with a background of impaired immunity, including malignancies, autoimmune diseases, and other immune disorders. Cytotoxic PTCL-NOS, is associated with mutations in epigenetic modifier genes and signaling pathways [36] and shows an activated cytotoxic phenotype [37]. They often fit into the PTCL-TBX21 subgroup and have poor prognosis [38].

In the workshop, 10 cases were submitted with the diagnosis of cytotoxic T-cell lymphoma. Nine cases represented examples of cPTCL-NOS (supplementary table 3). All 9 cases were EBV-negative. There was a slight predominance of male patients (M:F; 5:4) with a median age of 57 years (range 13–78 years). Three patients had a background of immune dysregulation. Histologically, the cases showed effacement of the nodal architecture, and the neoplastic cells were predominantly medium to large. In contrast to the other cases, the cells in case LYWS-1367 submitted by H Shao were small and without atypia. In addition, the Ki67 proliferation index was low, suggesting that it may represent a form of indolent PTCL [39]. This case also displayed aberrant positivity for CD20, a well-recognized phenomenon in mature T-cell malignancies [40]. Immunophenotypically, 5 cases were CD8+, 3 cases were CD4+/CD8+, and 1 case was CD4+. Of the cases tested, 3 expressed TCR alpha/beta, and 2 cases were TCR silent. Five out of 8 cases had an activated cytotoxic phenotype, and all 3 cases with available data were classified into the PTCL-TBX21 subtype. CD56 expression was rare with only 1 case positive. CD30 positivity was present in 5 out of 8 cases.

In agreement with the literature, NGS studies identified mutations in the epigenetic modifier genes in 5 out of 5 cases for molecular studies [20] (supplementary table 3). Information regarding the outcome was available in 6 of 9 cases. Case LYWS-1235 achieved complete remission, and three cases were in partial response after 8, 12, and 60 months of follow-up. Two patients died of lymphoma, LYWS-1200 after 6 months and case LYWS-1213 after 19 months from the diagnosis.

**Fig. 4:**
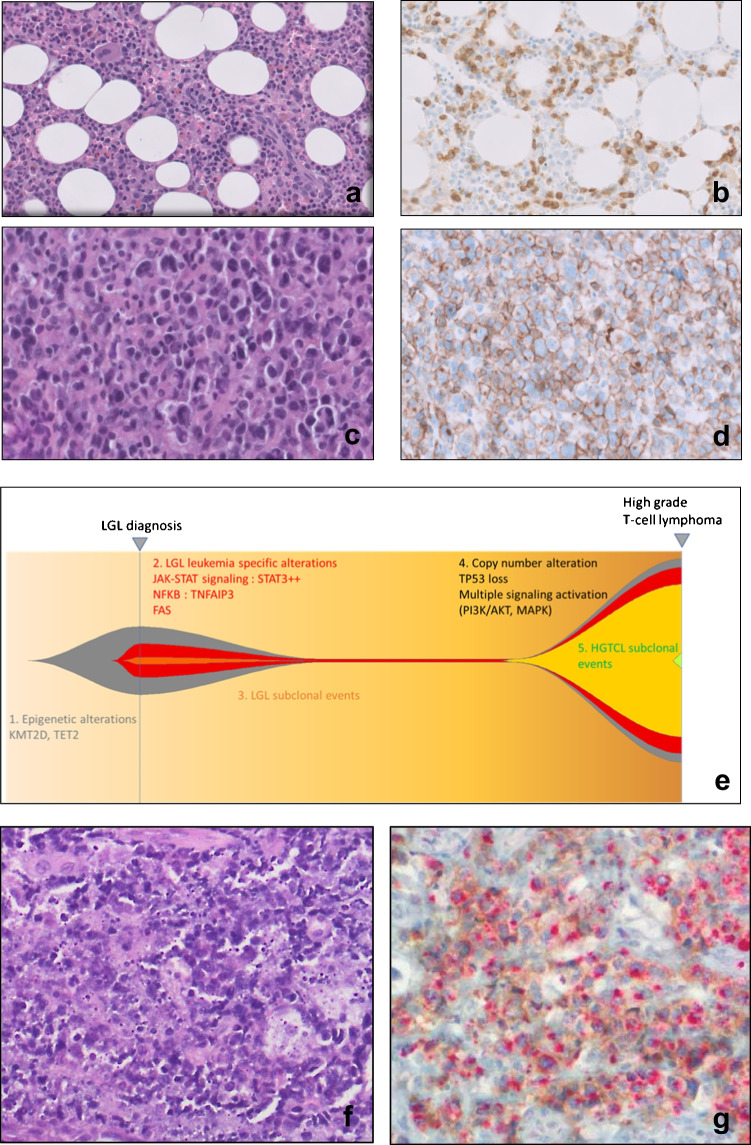
LGL transformation case (LYWS-1416 submitted by F. Gutierrez-Llamas). **a** The BM biopsy in this 78-year-old male shows interstitial infiltrate of small lymphocytes **b** expressing CD3. The lymphocytes are also positive for CD8 and Granzyme B. **c** The lymph node shows an atypical infiltrate of large cells (H&E), that are positive for **d** CD8, CD56, CD30, Granzyme B, and p53. Clonal analysis revealed the same TR gamma rearrangement in both samples. **e** NGS confirms the presence of the same TET2 mutations in both sites, without the STAT3 mutation and the presence of new mutations (JAK3 and KRAS) in the transformation sample (**f** and **g**). Case LYWS-1200 submitted by G Frigola displays the co-expression of cytotoxic and TFH markers in the same cells [double stain PD1 (brown) TIA-1 (red)]

The case of LYWS-1416, as submitted by F Gutierrez-Llamas, provides a remarkable illustration of large granular lymphocytic leukemia (LGL) transformation shedding light on the possibility of cPTCL-NOS progressing from an indolent T-cell leukemia. This phenomenon has been sparsely documented in existing literature, with only a handful of cases reported [6, 7]. The case highlights the clonal relationship between the two LPDs, and the whole exome sequencing analysis further suggested a hierarchical multi-hit evolution, with early epigenetic events possibly playing a significant role (Fig. 4). This case LYWS-1416 and another case of LGLL with transformation from the French LGLL registry have recently been published, and the authors discussed the pathogenesis of LGLL transformation [9].

The case LYWS-1200, submitted by G Frigola, is a unique example of a cPTCL-NOS, where the tumor cells co-expressed cytotoxic (granzyme B, TIA1, and perforin) and TFH (PD1, CXCL13 and BCL6) markers within the same cells. This can be clearly observed in Fig. 4, where the double stain for PD1 and TIA1 highlights this colocalization. The exact origin of these cells is unclear, but it is possible that they may represent distinct subtypes of TFH cells with cytotoxic activity [41–44].

The last case (LYWS-1462 submitted by D Dueñas) was a good example of T-prolymphocytic leukemia based on the history of the patient (a white blood cell count of 56.9 × 10^9^/L), morphology, and TCL-1 expression by IHC.

The most important messages gleaned from cPTCL-NOS cases in the workshop and recent literature are summarized as follow:

1. cPTCL-NOS, EBV negative, frequently present in a background of impaired immunity, similar to primary nodal-EBV-TNKL.
2. The majority of cases are subclassified into PTCL-TBX21 subtype based on CXCR3, TBX21, CCR4, and GATA3 expression pattern.
3. The mutational landscape of cPTCL-NOS, EBV negative, is similar to primary nodal-EBV-TNKL and is characterized by mutation of epigenetic modifiers, suggesting a potential role of CH in the lymphoma pathogenesis.

## Miscellaneous cytotoxic PTCL

The workshop received 4 cases of miscellaneous cytotoxic T-cell proliferations (supplementary table 4). Case LYWS-1131 from A Tzankov illustrated an unusual example of primary cutaneous gamma delta T-cell lymphoma that presented with a solitary cutaneous lesion. Case LYWS-1418 from J Coviello was a case of CD30+ large cell lymphoma with features of ALK-negative anaplastic large cell lymphoma (ALCL) and possible PAX5 expression in rare CD30+ tumor cells, raising the differential diagnosis of CHL with expression of T-cell markers. TR gene rearrangement performed by the panel revealed monoclonal rearrangement of TRB and TRG, confirming the diagnosis of ALK-negative ALCL. Case LYWS-1293 submitted by J Gao was a good example of EBV-negative ANKL. The case presented with HLH, systemic disease, NK-origin, and complex karyotype [45]. As reported in the literature, these cases are indistinguishable clinically and pathologically from EBV-positive ANKL. Case LYWS-1467 from E.I. Dvindenko was a difficult example of T-lymphoblastic lymphoma/leukemia (LBL) with maturation. Given the overlapping diagnostic features with indolent T-lymphoblastic proliferations, the positivity for LMO2 favors the diagnosis of T-LBL. LMO-2 has been described as a sensitive and specific marker for differentiating T-LBL from indolent T-lymphoblastic proliferations [46].

## Findings of the workshop

### Cytotoxic PTCL NOS associated with TFH lymphoproliferations

One of the interesting findings in this workshop was the remarkable, and unexpected, association of cPTCL-NOS with TFH lymphomas/LPDs. The workshop received six cases that illustrated the association of a TFH proliferation with a cytotoxic PTCL, both EBV-positive and negative (supplementary table 5 and supplementary table 5-mutations). Interestingly, three of them had a history of immune dysregulation (methotrexate treatment, hepatitis B, and rituximab-fludarabine cyclophosphamide for chronic lymphocytic leukemia (CLL)). cPTCL-NOS often occurs in patients who are immunocompromised, and this immune dysfunction likely contributes to lymphomagenesis [16, 20, 47]. In three of the cases, the initial LPD was the TFH lymphoma while in the other three cases, the LPD that presented first was the cPTCL-NOS. In five cases with available material for molecular studies, the TR gene rearrangement showed that the TFH and the cytotoxic proliferations were clonally unrelated.

Interestingly, two of the six TFH LPDs were clonal but the infiltration was focal without effacement of the nodal architecture. In these two cases, the abnormal clone was originally identified by flow cytometry analysis. One example of these 2 cases is case LYWS-1402 submitted by M Klimkowska. The patient had a background of immune dysregulation, given the history of CLL, and developed enlargement of an axillary lymph node in 2014. Flow cytometry analysis, IHC, and TR rearrangement were performed, and a TFH clonal proliferation was detected; the patient received corticosteroids and the symptoms improved. The panel agreed with the submitter that the morphological changes were insufficient to render a diagnosis of TFH lymphoma. In 2017, the patient presented with generalized lymphadenopathy and the excised lymph node displayed a diffuse infiltrate of large pleomorphic cells partially effacing the nodal architecture. The T-cell proliferation was positive for CD4, CD56, and perforin, and monoclonal for TR gene rearrangement. This case underscores the importance of flow cytometry, IHC, and molecular testing (TR and NGS) in identifying and characterizing a small population of TFH cells for accurate diagnosis. TFH proliferations can be challenging to diagnose as they may represent either a smoldering or an early manifestation of AITL [48]. Furthermore, expansions of reactive TFH cells can be seen in reactive lymphadenopathies and B-cell lymphomas, such as nodal and extranodal marginal zone lymphomas [49]. Unfortunately, material was not submitted for case LYWS-1402 (2014 lymph node) for additional NGS testing. The panel would support the diagnosis of TFH lymphoma for the clonal TFH proliferation in the 2014 lymph node biopsy if typical mutations of TFH lymphoma, such as RHOA mutation, could be demonstrated.

**Fig. 5:**
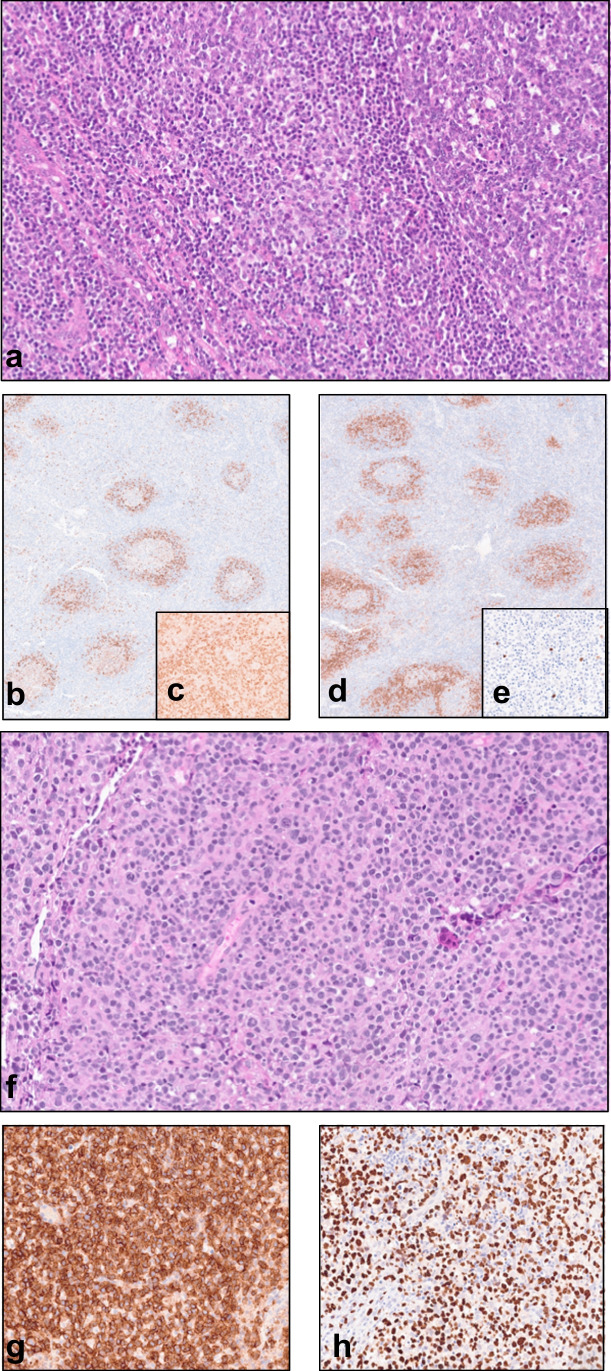
Case LYWS-1396, presented by L Wang, shows the association of a cytotoxic nodal EBV+ T and NK-cell lymphoma (primary nodal-EBV-NKTL) and an angioimmunoblastic T-cell lymphoma (AITL) in a 48-year-old female. The initial LN biopsy shows an AITL pattern 1 with reactive follicles (**a**). The tumor displays a T-follicular helper (TFH) phenotype with positive expression of PD1 (**b**, perifollicular pattern), expression of IDH2R172K (**c**), and CD10 (**d**). Scattered large B blasts are highlighted by EBER (**e**). The second lymphoma displays a diffuse proliferation of atypical medium-large cells (**f**). Immunohistochemistry shows positivity for CD8 (**g**) and EBER (**h**)

Case LYWS-1396 submitted by L Wang represented a case of primary nodal-EBV-TNKL occurring in the context of an untreated TFH lymphoma of angioimmunoblastic-type (AITL) with an indolent course spanning 6 years. This case highlights the potential for primary nodal-EBV-TNKL to develop presumably in the presence of immune dysfunction related to TFH proliferation/lymphoma and possibly aggravated by EBV reactivation, even in the absence of treatment [50]. In this case, the two lymphomas were clonally unrelated. Mutations in *TET2*, *RHOA*, and *IDH2* were present in the AITL. However, NGS did not detect mutations in the primary nodal-EBV-TNKL confirming further that these two neoplasms were not related [20] (Fig. 5).

### TBX21/GATA3 subtypes

The definition of PTCL-NOS as a mature T-cell lymphoma not meeting the criteria for other specific entities remains unchanged in the 2022 ICC [5] and the 5th WHO classification [4]. Gene expression profiling studies have identified two major molecular subgroups: one overexpressing TBX21 and the other overexpressing GATA3 [51]. Compared to PTCL-TBX21, PTCL-GATA3 has a worse prognosis, with higher genomic complexity, including 17p del (TP53), 9p del (CDKN2A), and 10p del (PTEN), and gains of *STAT3* and *MYC*. PTCL-TBX21 has a better prognosis, less genomic complexity, and a higher frequency of mutations involving epigenetic modifying genes [37]. An immunohistochemical algorithm using antibodies to TBX21, CXCR3, GATA3, and CCR4 has been proposed to stratify PTCL-NOS into TBX21 and GATA3 subgroups [38].

The panel performed the IHC algorithm [38] in 9 of 21 cases of cytotoxic PTCL, both EBV+ and EBV−, submitted to the workshop with available material. The results are detailed in Table 2. Seven out of the 9 cases analyzed were classified into the TBX21-subtype, one case corresponded to the GATA3-subtype, and one case was unclassifiable. Our findings are in line with previous reports describing the expression of cytotoxic markers to be more frequently associated with PTCL-TBX21 compared with PTCL-GATA3 [37, 38].

**Table 2:** Immunophenotypic features of cytotoxic PTCL-NOS based on the workshop cases and cases published in recent literature (Nicolae et al. and Wai et al.) [20, 16]

	Workshop cases	Nicolae et al. [20]	Wai et al. [16]
CD8	13/21 (62%)	28/54 (45%)	17/25 (68%)
TCRβF1	9/12 (75%)	17/36 (47%)	12/25 (48%)
AC phenotype	15/18 (83%)	45/54 (98%)	NA
TBX21 phenotype	7/9 (78%)	36/38 (95%)	NA
CD30 positive	7/18 (39%)	36/53 (68%)	NA
CD56 positive	4/21 (19%)	4/44 (9%)	6/24 (25%)

*AC* activated cytotoxic, *NA* not available

### Mutational landscape of nodal cytotoxic PTCL, EBV-positive, and EBV-negative

The panel analyzed the mutational profile of cytotoxic PTCL, EBV-positive, and EBV-negative from the workshop and added the information of 2 recent studies from Wai CMM and Nicolae A et al. [16, 20]. A total of 61 cases were analyzed. Like primary nodal-EBV-TNKL, the genetic landscape showed frequent mutations in epigenetic modifiers (44/61, 72%), followed by mutations in TCR (15/61, 25%) and JAK/STAT (14/61, 23%) signaling pathway, irrespective of the EBV status (Fig. 2). Co-occurence of *TET2* and *DNMT3A* mutations were present in 14 of 61 cases (23%) (Fig. 2B).

The mutational profile of PTCL-NOS is enriched in *TET2* and *DNMT3A* mutations. Mutations in these two genes co-occurred, suggesting an oncogenic cooperation, as observed in TFH lymphomas [52]. The loss of 5-hydroxymethylcytosine due to *TET2* mutation and DNA hypomethylation because of *DNMT3A* loss in critical target genes may act synergistically in promoting lymphomagenesis [53, 54]. While *TET2* mutation occurs at near-equal frequencies in PTCL-GATA3 and PTCL-TBX21, *DNMT3A*, *TET*,*1* and *TET3* mutations were more commonly detected in PTCL-TBX21 [37].

Herek TA et al. recently reported the association of *DNMT3A* mutations with PTCL-TBX21 subtype and demonstrated that the R882 variant particularly correlated with cytotoxic differentiation and inferior clinical outcome [55]. One of 3 cases of primary nodal-EBV-TNKL with *DNMT3A* mutation, LYWS-1207, submitted by K Ofori, demonstrated the *DNMT3A*^R882P^ variant but displayed the PTCL-GATA3 phenotype based on IHC (TBET−, CXCR3−, GATA3+, CCR4−). The distinct prevalence of the *DNMT3A*^R882H/C^ variant in PTCL-NOS compared to AITL is intriguing and requires further investigations [55].

### Role of clonal hematopoiesis (CH)

**Fig. 6.**
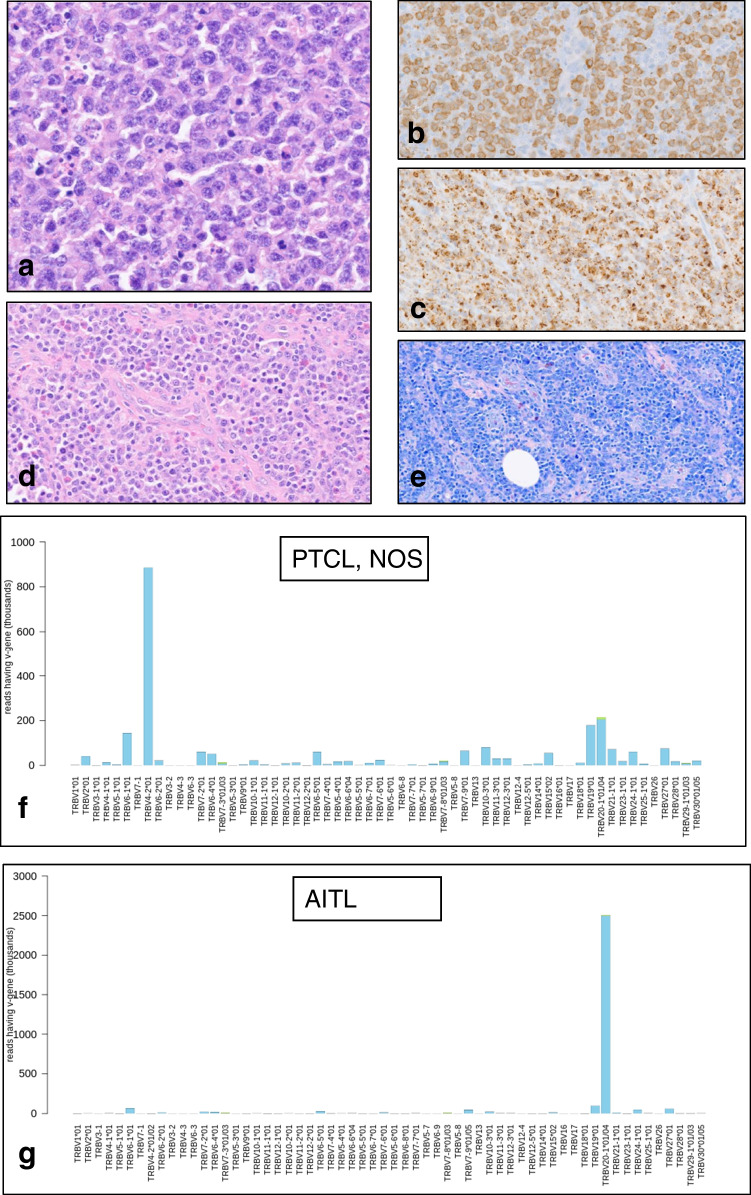
Case LYWS-1094, presented by A. Volgelsberg, describes the divergent evolution of two clonally unrelated T-cell lymphomas from clonal hematopoiesis. A 71-year-old woman presented with enlarged cervical LNs. The biopsy shows diffuse infiltration of large cells (**a**). Immunohistochemistry illustrates positivity for CD3 (**b**), TIA-1 (**c**), CD8 (not shown), and βF1 (not shown). The patient received CHOP therapy and achieved complete remission. Five months later, the patient relapsed and the lymph node reveals a mixed infiltration of lymphoid cells (**d**) and proliferation of high endothelial venules (**e**). TR beta sequencing of both lymphomas confirms the presence of distinctly different rearrangements (**f** and **g**)

CH is common among patients with lymphoma, and its frequency increases with age [56]. TFH lymphomas frequently harbor *TET2* and *DNMT3A* mutations, and identical mutations have been identified in both the malignant T-cells and the myeloid component of patients, suggesting a common ancestral clone with subsequent divergent evolution [57]. Notably, our analysis of 61 cytotoxic PTCL cases, EBV+ and EBV−, from the workshop and 2 recent studies [16, 20] revealed 44 out of 61 cases (72%) with mutations of epigenetic modifier genes. Co-occurrence of *TET2* and *DNMT3A* mutations were present in 23%. These findings suggest a potential role of CH in the pathogenesis of cytotoxic PTCL. In addition, one workshop case (LYWS-1094 submitted by A Vogelsberg) nicely illustrated the role of CH in the development of PTCLs with different phenotypes and origin from a common progenitor. This case provided evidence for a divergent evolution of two clonally-unrelated T-cell lymphomas (cPTCL-NOS and AITL) originating from a common progenitor, which shared the same mutations in *TET2* and *DNMT3A* (Fig. 6). These mutations were detected in the BM biopsies, which were morphologically and molecularly negative for lymphoma, suggesting that CH is not only a precursor of AITL but also a precursor of cPTCL-NOS. Notably, a recent case report by Attygalle et al. described two cases showing parallel evolution of two distinct and neoplastic lymphoid proliferations from a common *TET2-DNMT3A* mutated hematopoietic progenitor cell population [50]. It remains uncertain if the other 5 workshop cases showing the association between TFH lymphoma/LPD and cPTCL-NOS are derived from a common progenitor (Supplementary table 5-mutations).

## Conclusion

Primary nodal-EBV-TNKL is a rare and aggressive lymphoma characterized by T-cell lineage, lack of nasal involvement, low genomic instability, frequent loss of 14q11.2, and upregulation of immune pathways, NFκB and PD-L1. Primary nodal TNKL can occasionally involve the tonsils/Waldeyer’s ring and be misdiagnosed as upper aerodigestive tract involved by ENKTL. In addition, EBV+ T/NK LPD involving extranodal sites in children and adults, such as SEBVTCL and ANKL, can occasionally show prominent LN involvement either at disease presentation or following transformation from CAEBV disease and should not be diagnosed as primary nodal-EBV-TNKL, which affects elderly patients and often associated with immunosuppression.

Based on the workshop cases and recent literature, the mutational landscape of primary nodal-EBV-TNKL is similar to cPTCL-NOS and is characterized by frequent mutation of epigenetic modifiers, such as *TET2* and *DNMT3A*, and JAK/STAT pathway genes suggesting a potential role of CH and JAK/STAT pathway in the pathogenesis of cytotoxic PTCL. Whether primary nodal-EBV-TNKL represents an EBV-positive counterpart of cPTCL-NOS, or a distinct entity requires further study.

cPTCL-NOS are associated with settings of immune dysregulation, derive from mature T lymphocytes, commonly alpha/beta, display an activated cytotoxic phenotype, and the majority are of PTCL-TBX21 subtype. These cases show frequent mutations in epigenetic modifier genes. The cases submitted to the workshop underline the need for close examination of the T-cell proliferations to identify and better characterize the TFH and cytotoxic proliferations. The workshop cases have not only identified a novel association between TFH LPD/lymphoma and cPTCL-NOS, but also highlighted the potential role of CH in the development of neoplastic proliferations of different phenotypes. More cases and studies are warranted to further understand this important observation.

### Supplementary information

ESM 1 (XLSX 25 kb)

## Data Availability

Not applicable.
